# Supplementary material for: Temporal stability of fMRI in medetomidine-anesthetized rats
Source: Sci Rep. 2019 Nov 13;9:16673. doi: 10.1038/s41598-019-53144-y (PMC6853937; doi:10.1038/s41598-019-53144-y)
Supplement: Supplementary file 1 — Supplementary Material [file 41598_2019_53144_MOESM1_ESM.docx]

Supplementary Material

# Temporal stability of fMRI in medetomidine-anesthetized rats

Nikoloz Sirmpilatze^*,1,2,3,4^, Jürgen Baudewig^1^, Susann Boretius^1,2,3,4^.

1. Functional Imaging Laboratory, German Primate Center, Göttingen, Germany
2. Georg-August University of Göttingen, Göttingen, Germany
3. International Max Planck Research School for Neurosciences, Göttingen, Germany
4. DFG Research Center for Nanoscale Microscopy and Molecular Physiology of the Brain (CNMPB), Göttingen, Germany

- *Corresponding author:* Nikoloz Sirmpilatze, Deutsches Primatenzentrum GmbH, Kellnerweg 4, 37077 Göttingen, Germany***.*** NSirmpilatze@dpz.eu

*Alternative correpondence:* Susann Boretius, Deutsches Primatenzentrum GmbH, Kellnerweg 4, 37077 Göttingen, Germany. SBoretius@dpz.eu

# Table of contents

| **S1** | Table | (Dex)medetomidine administration protocols used by studies performing BOLD fMRI in rats (p. 2) |
| --- | --- | --- |
| **S2** | Figure | Custom-built MRI-compatible rat bed with head holder (p. 3) |
| **S3** | Figure | Heart rate of individual animals (p. 4) |
| **S4** | Figure | Respiratory rate of individual animals (p. 5) |
| **S5** | Figure | Example fMRI images (p. 6) |
| **S6** | Figure | Quantification of head motion (p. 7) |
| **S7** | Figure | Peak ΔBOLD and global RSFC of individual animals (p. 8) |
| **S8** | Figure | Areas activated by electrical forepaw stimulation (EFS): second-level group analysis (p. 9) |
| **S9** | Table | ANOVA tables (p. 10) |
| **S10** | Table | Least Square Means tables (p. 11) |
| **S11** | Table | Pair-wise comparisons for Peak ΔBOLD (p. 12) |
| **S12** | Table | Pair-wise comparisons for Global RSFC (p. 13) |
|  |  |  |

## Supplementary Table S1. (Dex)medetomidine administration protocols used by studies performing BOLD fMRI in rats

SC: subcutaneous; IV: intravenous; IP: intraperitoneal; M: male; F: female; SD: Sprague-Dawley; LH: Lister-Hooded; LE: Long-Evans.

| Drug | Route | Bolus dose  (mg/kg) | Infusion rate  (mg/kg/h) | Rat  sex | Rat strain | Study |
| --- | --- | --- | --- | --- | --- | --- |
| Medetomidine | SC | 0.05 | 0.1 | M | Wistar | Weber et al., 2006^1^ |
|  | SC | 0.05 | 0.1 | M | Wistar | Weber et al., 2008^2^ |
|  | SC | 0.05 | 0.1 | M | SD | Zhao et al., 2008^3^ |
|  | IV | - | 0.1 | - | SD | Pawela et al., 2008^4^ |
|  | IV | - | 0.1 / 0.3 / 0.1 to 0.3 | M | SD | Pawela et al., 2009^5^ |
|  | SC | 0.05 | 0.1 | M | Wistar | Seehafer et al., 2010^6^ |
|  | SC | 0.05 | 0.1 | M | SD | Williams et al., 2010^7^ |
|  | SC | 0.05 | 0.1 | M | Wistar | Angenstein et al., 2010^8^ |
|  | SC | 0.05 | 0.1 | M | Wistar | Airaksinen et al., 2010^9^ |
|  | SC | 0.05 | 0.1 | M | SD | Jonckers et al., 2011^10^ |
|  | SC | 0.5 | 0.1 | - | - | Majeed et al., 2011^11^ |
|  | SC | 0.05 | 0.1 | M | Wistar | Airaksinen et al., 2012^12^ |
|  | SC | 0.05 | 0.1 | M | Wistar | Krautwald and Angenstein, 2012^13^ |
|  | IV | 0.3 | - | M | SD | Ciobanu et al., 2012^14^ |
|  | IP | 0.05 | 0.1 / 0.2 / 0.3 | M | Wistar | Nasrallah et al., 2012^15^ |
|  | SC | 0.05 | 0.1 | M | Wistar | Kalthoff et al., 2013^16^ |
|  | SC | 0.07 | 0.14 | M | SD | Schwarz et al., 2013^17^ |
|  | SC | 0.05 | 0.1 | M | Wistar | Angenstein et al., 2013^18^ |
|  | SC | 0.05 | 0.1 | M | LH | Sekar et al., 2013^19^ |
|  | IV | 0.3 | - | M | SD | Uhrig et al., 2014^20^ |
|  | IP | 0.05 | 0.1/ 0.2/ 0.3 | M | Wistar | Nasrallah et al., 2014^21^ |
|  | SC | 0.2 | 0.1 | M | SD | D’Souza et al., 2014^22^ |
|  | SC to IV | 0.05 | 0.1 | M | SD | Duffy et al., 2015^23^ |
|  | SC | 0.05 | 0.1 | M | SD | Sierakowiak et al., 2015^24^ |
|  | IP | 0.05 | 0.1 | M | Wistar | Nasrallah et al., 2016^25^ |
|  | IV | - | 0.1 | M | Wistar | Paasonen et al., 2016^26^ |
|  | SC | 0.05 | 0.1 | M | Wistar | Scherf and Angenstein, 2017^27^ |
|  | SC | 0.04 | 0.05 | F | Fisher | Albers et al., 2018^28^ |
|  | IV | 0.05 | 0.1 | M | SD | Wang et al., 2018^29^ |
|  | IV | - | 0.1 | M | SD | Shatillo et al., 2018^30^ |
|  | IV | - | 0.1 | M | Wistar | Paasonen et al., 2018^31^ |
|  |  |  |  |  |  |  |
| Dexmedetomidine* | IV | 0.05 | 0.05 | M | SD | Fukuda et al., 2013^32^ |
|  | SC | 0.025 | 0.05 to 0.15 | M | SD | Pan et al., 2013^33^ |
|  | SC | 0.025 | 0.05 | M | LE | Chao et al., 2014^34^ |
|  | SC | 0.025 | 0.05 to 0.15 | M | SD | Magnuson et al., 2014a^35^ |
|  | SC | 0.025 | 0.05 to 0.15 | M | SD | Magnuson et al., 2014b^36^ |
|  | SC | 0.05 | 0.1 | - | - | Li et al., 2014^37^ |
|  | SC | 0.05 | 0.1 | M | SD | Thompson et al., 2014a^38^ |
|  | SC | 0.025 | 0.05 | - | SD | Thompson et al., 2014b^39^ |
|  | SC | 0.025 | 0.05 to 0.15 | M | SD | Medda et al., 2016^40^ |
| * Dexmedetomidine is the active ingredient of medetomidine, and has twice its potency; it is typically used at half the dosage. | | | | | | |


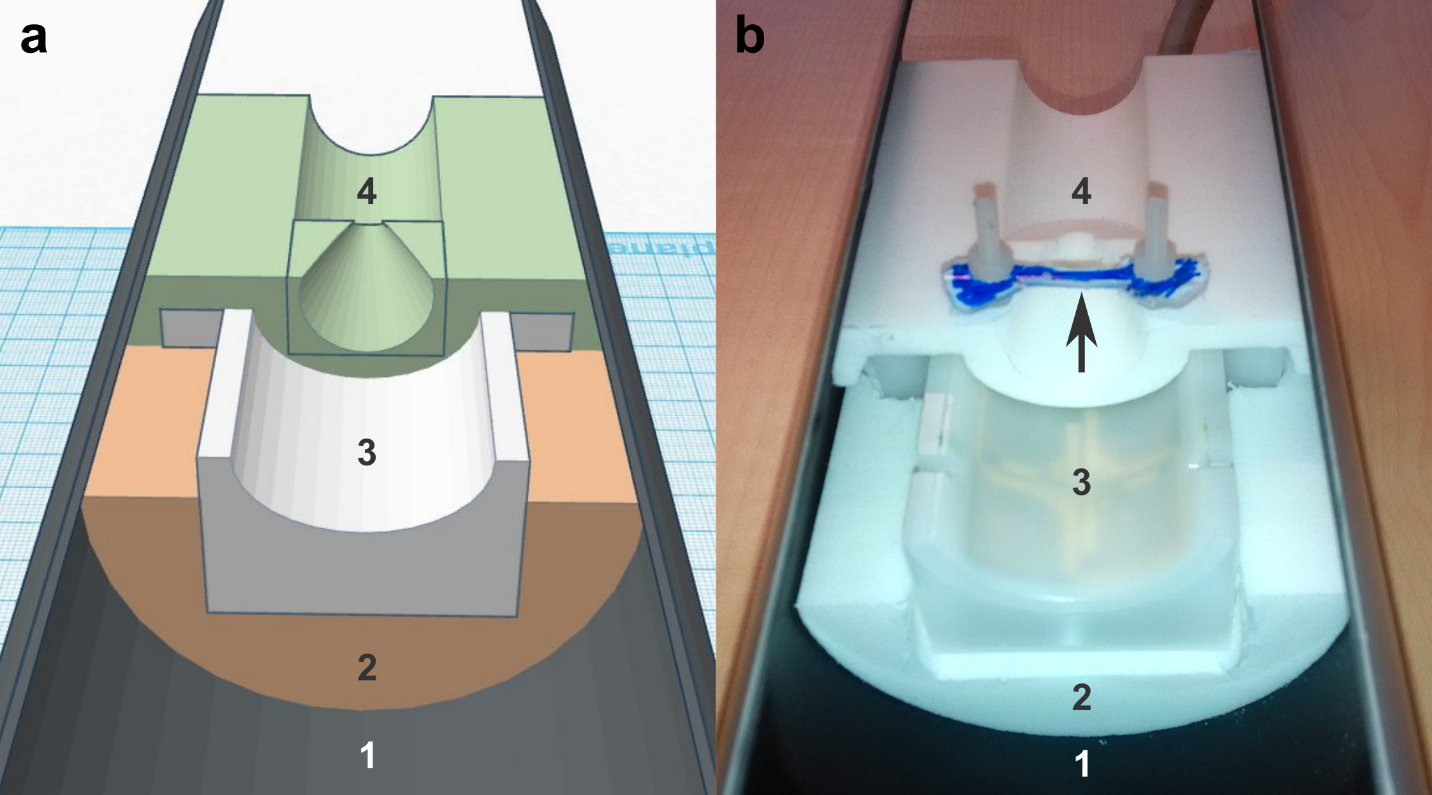


## Supplementary Figure S2. Custom-built MRI-compatible rat bed with head holder

A 3D model **(a)** and a photograph **(b)** of the rat cradle and head holder used during fMRI experiments. A plastic tube (1)—1 m long and 70 mm in diameter—serves as the base for the cradle. The top part of the tube is cut out (cord length of 55 mm) to accommodate access to the rat and the equipment. The rat brain 4-channel coil array (3) is stably positioned in the tube with the help of a 3D-printed adaptor piece (2). Another 3D-printed piece (4) fits on top of the coil array, providing a conic cavity. This cavity is designed to fit the rat’s snout (with a face mask), as the animal is lying supinely in the tube. The back of the rat’s head is pressed against the coil array and fixed with the help of a removable plastic bite bar (arrow), the height of which is adjustable via plastic screws. The entire setup can be inserted into the center of the volume resonator (inner diameter of 86 mm).


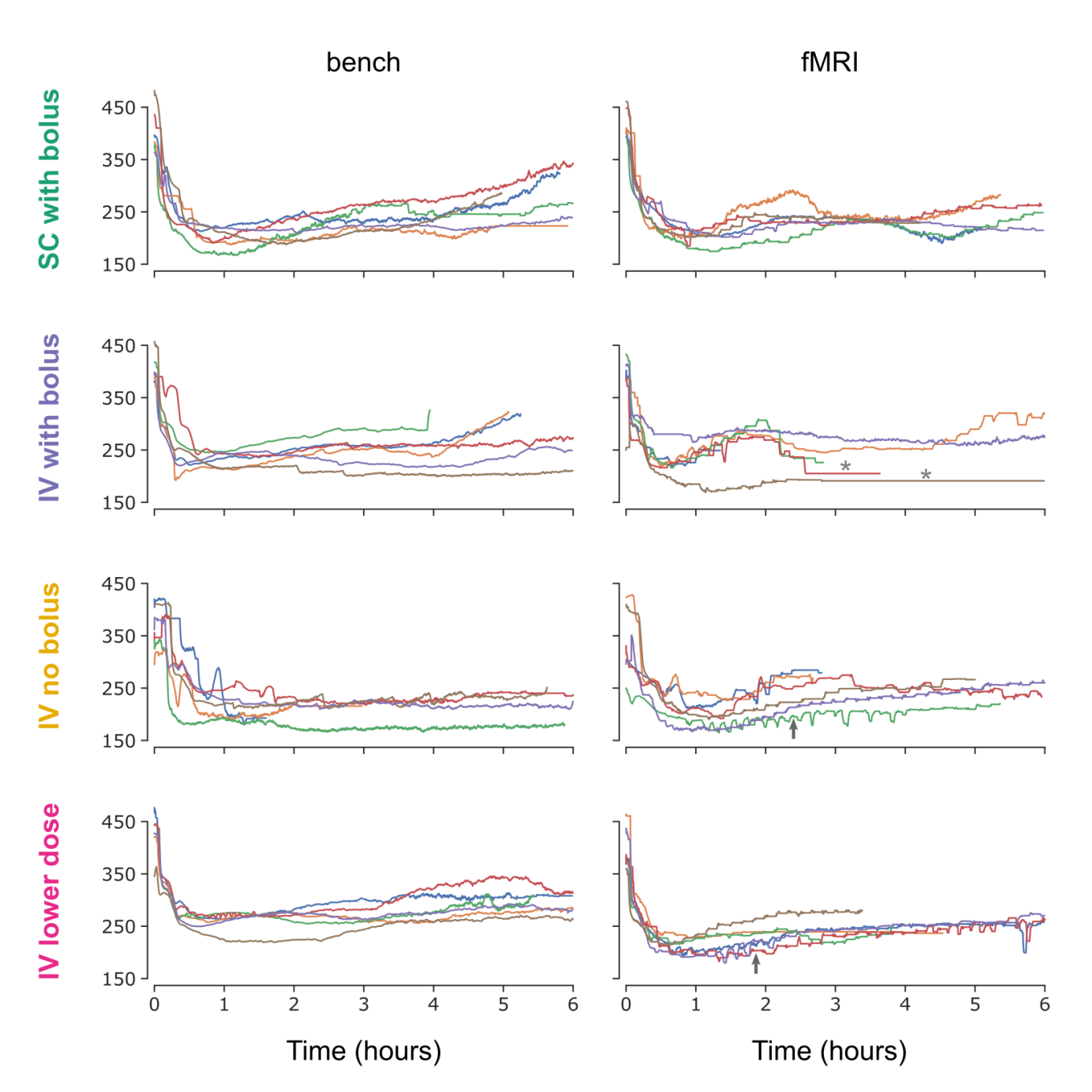


## Supplementary Figure S3. Heart rate of individual animals

Individual-rat heart rate traces (in beats per minute—bpm) are shown for all anesthesia sessions, bench and fMRI. The traces are preprocessed according to the procedure described in the manuscript’s methods section for “Anesthesia and monitoring”. Low-amplitude oscillations can be observed in some traces acquired during fMRI sessions (see arrows for examples). These do not represent real heart rate oscillations, but rather reflect the artefacts introduced during fMRI acquisition and the procedures followed for their removal. The straight lines that appear in a few traces (see asterisks) result from linearly interpolating over long periods of faulty data.


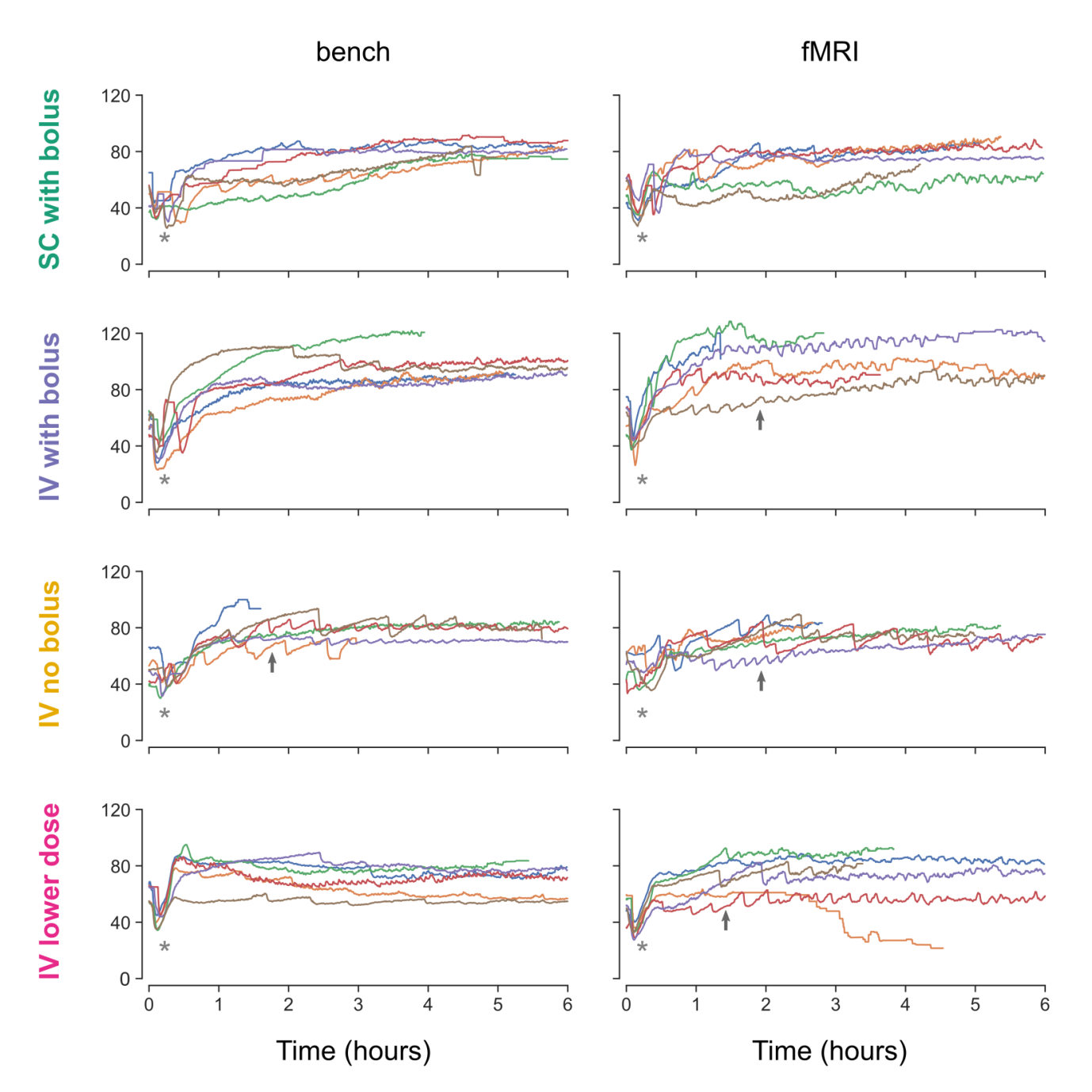


## Supplementary Figure S4. Respiratory rate of individual animals

Individual-rat respiratory rate traces (in breaths per minute—bpm) are shown for all anesthesia sessions, bench and fMRI. The traces are preprocessed according to the procedure described in the manuscript’s methods section for “Anesthesia and monitoring”. The observed dip in the initial 15 minutes of the recording (asterisks) corresponds to the overlap period between medetomidine and isoflurane, and most likely results from medetomidine enhancing isoflurane-mediated respiratory depression. Respiratory rates recover after isoflurane discontinuation and reach a steady-state level by the end of the first hour. Interestingly, the steady-state respiratory rate fluctuates quasi-periodically in many animals (see arrows for examples), in both bench and fMRI sessions, and across all medetomidine protocols. The amplitude and time-scale of these fluctuations vary among individual animals.


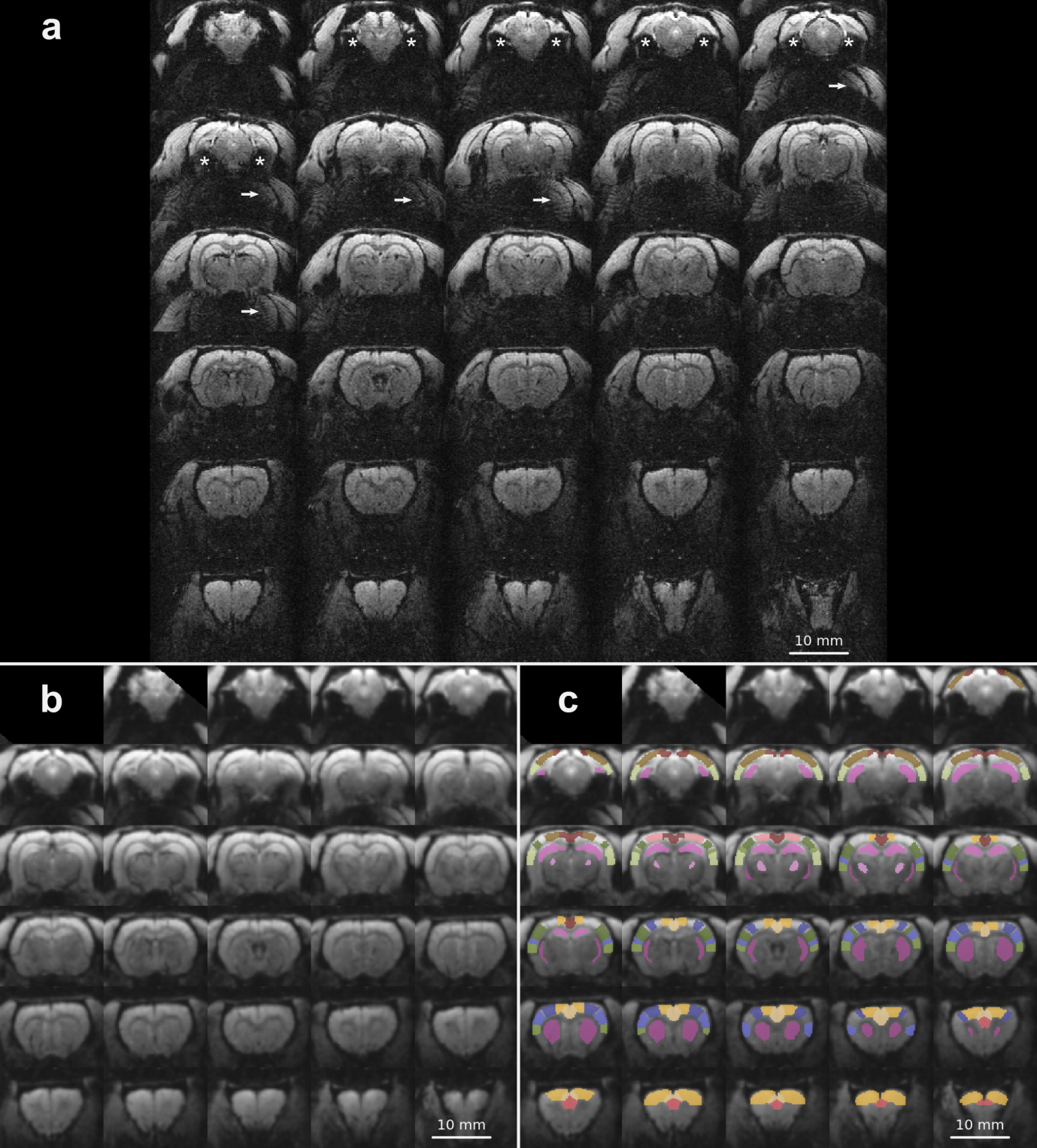


## Supplementary Figure S5. Example fMRI images

The middle volume of an example fMRI time-series **(a)**, acquired with a single-shot gradient-echo Echo Planar Imaging (EPI) sequence. The same volume, preprocessed and normalized to the common template space, is shown in **(b)**. The regions-of-interest (ROIs) used for resting-state functional connectivity analysis are overlaid on the preprocessed volume in **(c)**. Some typical EPI artifacts can be seen in the raw volume **(a)**, like susceptibility-induced signal drop-outs (asterisks) and EPI ghosts (arrows). However, the ROIs were restricted to areas unaffected by these artifacts **(c)**.


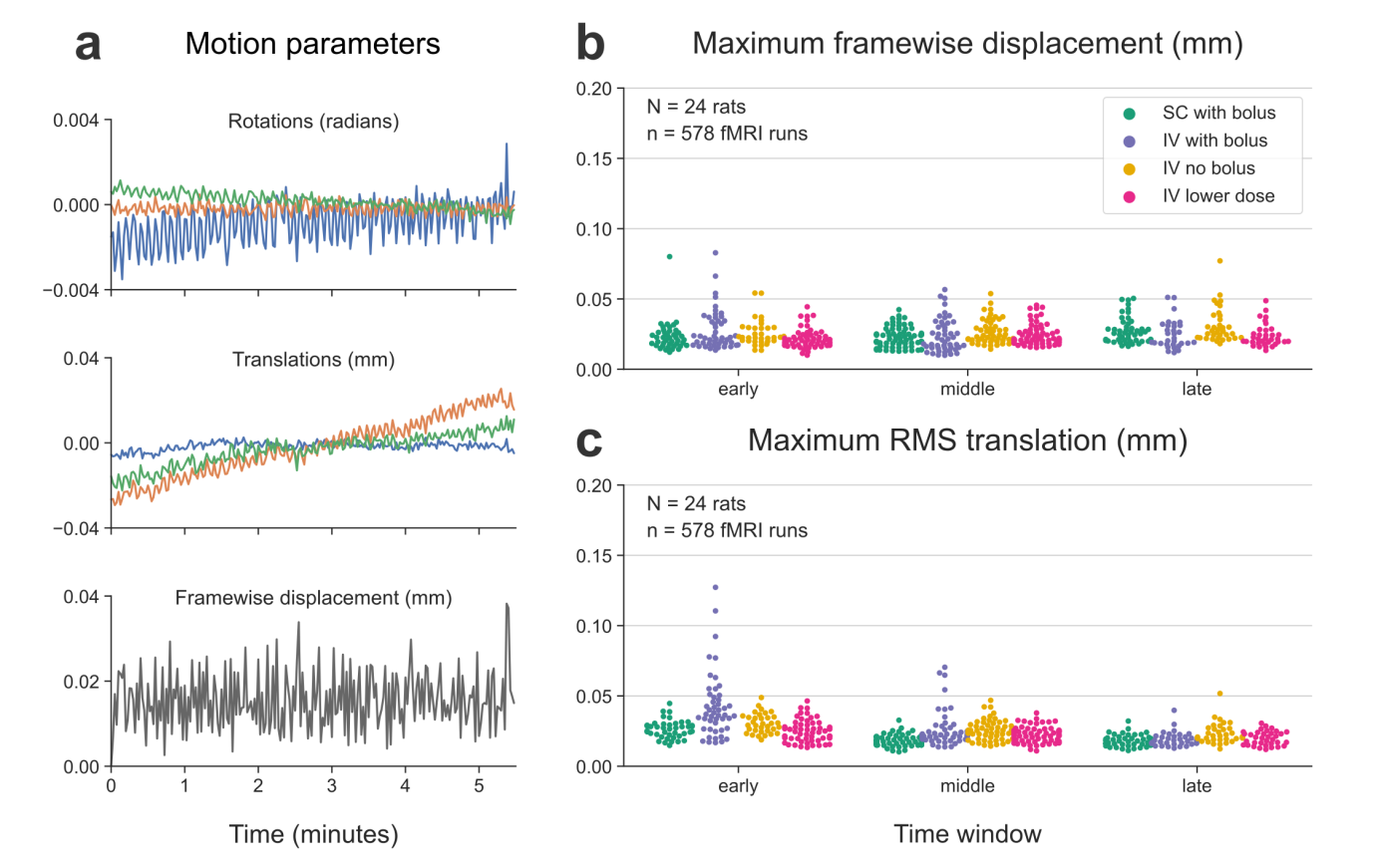


## Supplementary Figure S6. Quantification of head motion

Motion was estimated for all fMRI runs via the *antsMotionCorr.sh* script (part of Advanced Normalization Tools software—ANTs). Specifically, each volume of an fMRI run was spatially registered to the middle volume using a rigid body transform. This transform is defined by 6 parameters—3 rotations and 3 translations (shown in **(a)** for an example fMRI run)—which were used to extract two commonly reported measures of head motion: framewise displacement and root-mean-square (RMS) translation. Framewise displacement was computed according to Power et al. 2012^41^, using a sphere with a 5 mm radius for converting rotational angles to displacements (a 50 mm radius is used for humans). The computation of RMS translation (square root of x^2^ + y^2^ + z^2^, where x, y, and z are the 3 translation parameters) involves no such conversion. The maximum values of these two measures across all fMRI runs are shown in **(b)** and **(c)**.

^
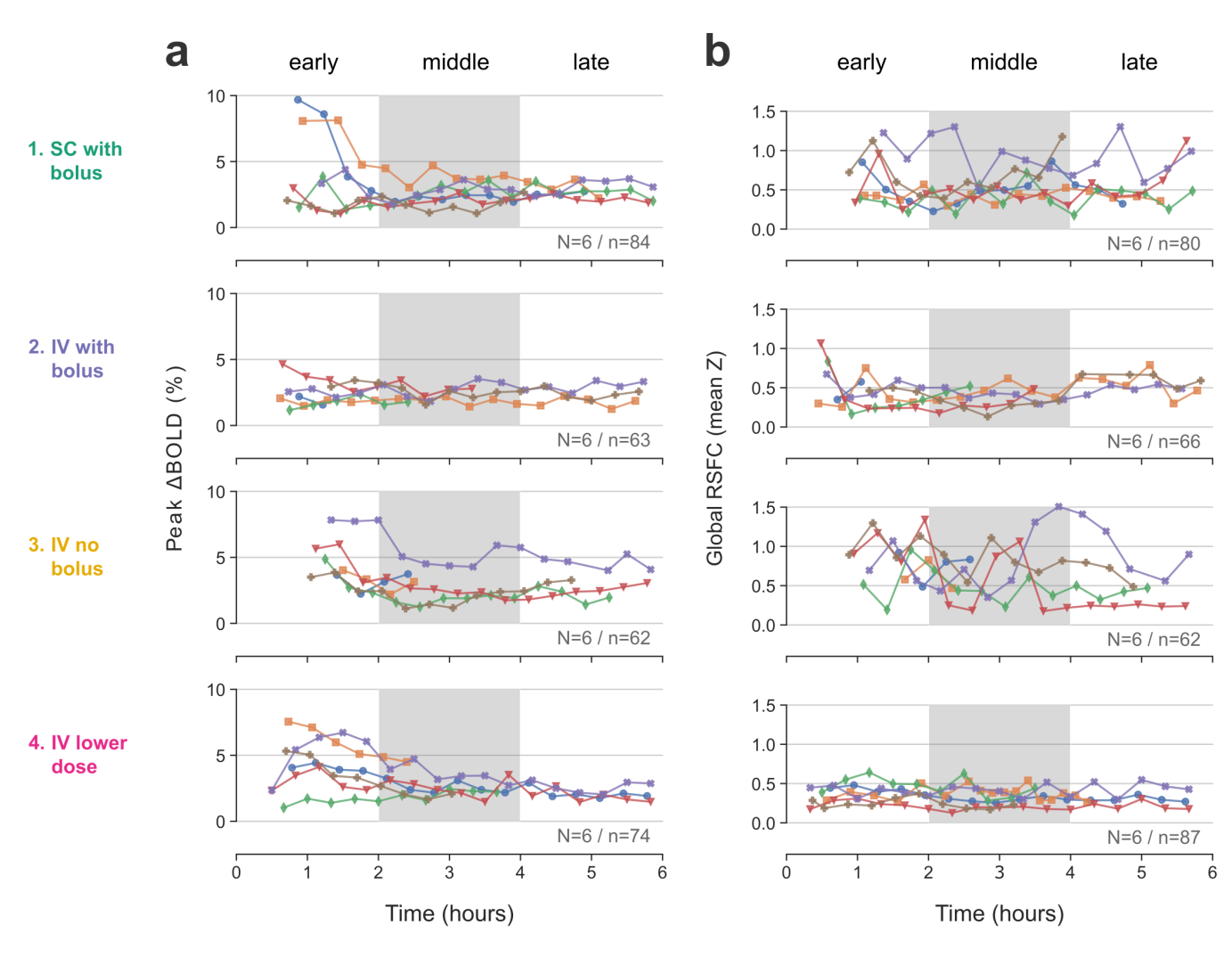
^

## Supplementary Figure S7. Peak ΔBOLD and global RSFC of individual animals

**(a)** The peak stimulus-evoked % signal change (peak ΔBOLD) is plotted for all EFS-fMRI runs across time. Each rat’s peak ΔBOLD time course is plotted as a separate trace. In **(b)**, the same type of plot is shown for the mean pair-wise correlation (global RSFC). These plots also demonstrate the binning of fMRI runs into three time-windows (early, middle, and late) for statistical analysis. N: number of rats; n: number of fMRI runs.

##
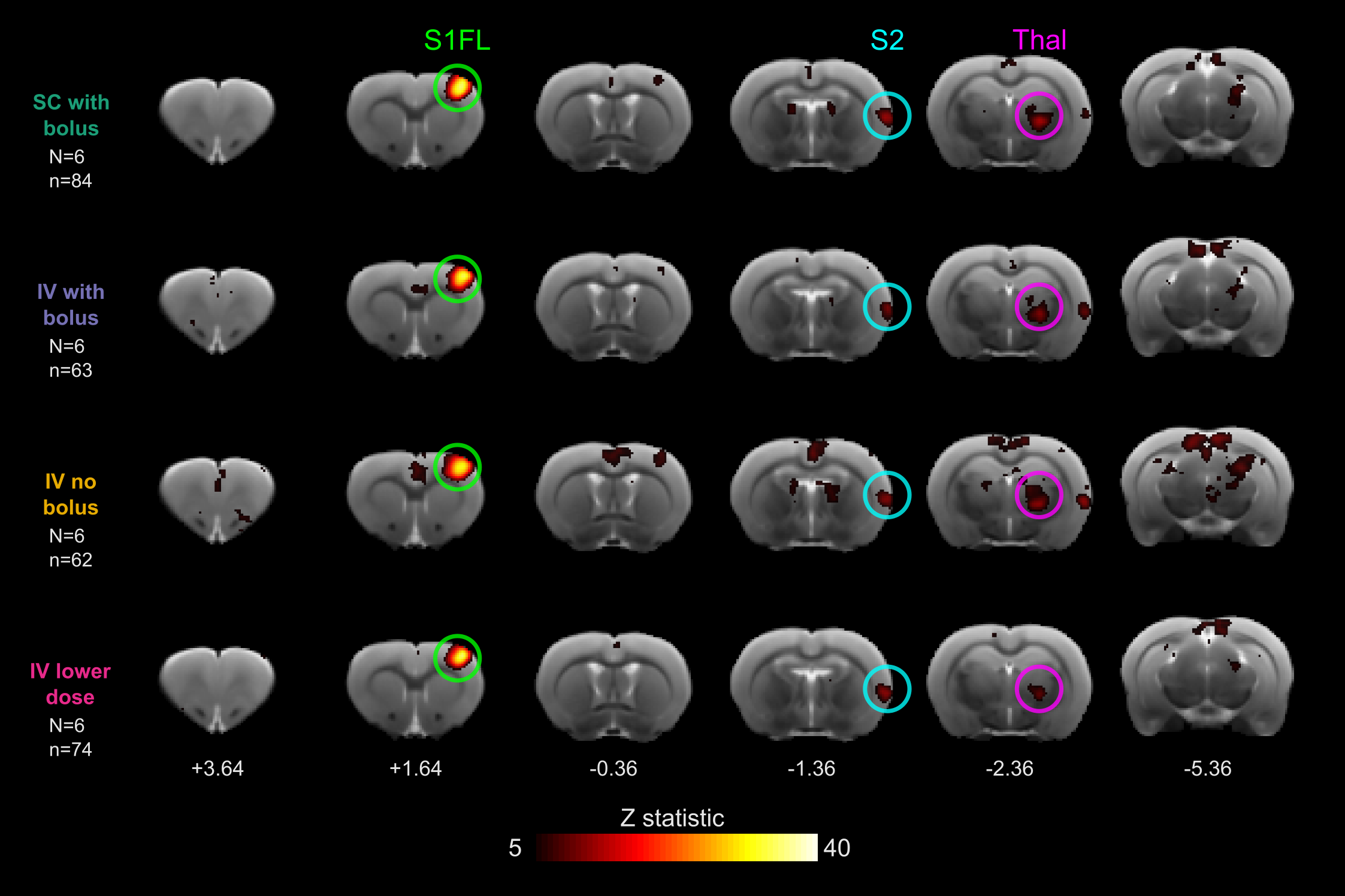


## Supplementary Figure S8. Areas activated by electrical forepaw stimulation (EFS): second-level group analysis

For each medetomidine protocol the corresponding first-level fMRI analysis results were pooled into a second-level fixed-effects analysis to compute the mean group effect (FMRI Expert Analysis Tool, version 6.00, part of FSL). The resulting statistical maps were masked for brain and thresholded non-parametrically using maximum height thresholding based on Gaussian random field theory, with a (corrected) significance threshold of p = 0.05. The thresholded maps are shown overlaid on a T2-weighted structural study template. The approximate rostral-caudal position of each slice is given in mm relative to the bregma (based on the Paxinos-Watson rat brain atlas). Strong activation in the forelimb region of the somatosensory cortex (S1FL) is found in all protocols, with weaker activations in the secondary somatosensory cortex (S2), and the thalamus (Thal). Active clusters can also be found in cingulate and retrosplenial cortices, and in the brainstem. N: number of rats; n: number of fMRI runs.

## Supplementary Table S9. ANOVA tables

Type III Analysis of Variance Tables with Satterthwaite's method (output of the *anova* function from the *lmerTest* R package). “Protocol” refers to the medetomidine administration protocol (1-4), while “Window” refers to the time-window (early, middle, or late). Significant effects (p<0.05) are shaded in grey.

| **Peak ΔBOLD** | | | | | | |
| --- | --- | --- | --- | --- | --- | --- |
|  | Sum Sq. | Mean Sq. | NumDF | DenDF | F value | Pr(>F) |
| Protocol | 2.648 | 0.883 | 3 | 24.341 | 1.055 | 0.3864 |
| Window | 63.017 | 31.508 | 2 | 263.390 | 37.650 | **<0.0001** |
| Protocol:Window | 21.815 | 3.636 | 6 | 263.252 | 4.345 | **0.0003** |
|  |  |  |  |  |  |  |
| **Global RSFC** | | | | | | |
|  | Sum Sq. | Mean Sq. | NumDF | DenDF | F value | Pr(>F) |
| Protocol | 0.807 | 0.269 | 3 | 25.602 | 7.241 | **0.0011** |
| Window | 0.382 | 0.191 | 2 | 279.816 | 5.146 | **0.0064** |
| Protocol:Window | 0.759 | 0.126 | 6 | 279.719 | 3.405 | **0.0029** |

## Supplementary Table S10. Least Square Means tables

Estimated Least Squares Means for all levels of protocol, time-window, and their combinations (output of the *ls_means* function from the *lmerTest* R package). A: early (1 – 2 h); B: middle (2 – 4 h); C: late (4 – 6 h); DF: degrees of freedom with Satterthwaite's method; lower and upper: bounds of the 95% confidence interval.

| **Peak ΔBOLD** | | | | | | |
| --- | --- | --- | --- | --- | --- | --- |
|  | Estimate | Std. Error | DF | t value | lower | upper |
| Protocol1 | 2.951 | 0.393 | 22.818 | 7.513 | 2.138 | 3.764 |
| Protocol2 | 2.326 | 0.408 | 25.682 | 5.706 | 1.487 | 3.164 |
| Protocol3 | 3.299 | 0.403 | 25.065 | 8.183 | 2.469 | 4.129 |
| Protocol4 | 3.074 | 0.398 | 23.943 | 7.723 | 2.252 | 3.895 |
| WindowA | 3.635 | 0.214 | 32.281 | 16.950 | 3.198 | 4.072 |
| WindowB | 2.610 | 0.210 | 29.636 | 12.405 | 2.180 | 3.040 |
| WindowC | 2.492 | 0.224 | 37.402 | 11.120 | 2.038 | 2.946 |
| Protocol1:WindowA | 3.645 | 0.426 | 31.648 | 8.546 | 2.776 | 4.514 |
| Protocol2:WindowA | 2.427 | 0.427 | 31.552 | 5.683 | 1.557 | 3.297 |
| Protocol3:WindowA | 4.408 | 0.444 | 36.844 | 9.937 | 3.509 | 5.307 |
| Protocol4:WindowA | 4.061 | 0.418 | 29.272 | 9.712 | 3.206 | 4.916 |
| Protocol1:WindowB | 2.521 | 0.409 | 26.713 | 6.169 | 1.682 | 3.360 |
| Protocol2:WindowB | 2.234 | 0.436 | 33.036 | 5.127 | 1.347 | 3.120 |
| Protocol3:WindowB | 2.721 | 0.421 | 29.898 | 6.457 | 1.860 | 3.581 |
| Protocol4:WindowB | 2.966 | 0.417 | 28.973 | 7.108 | 2.113 | 3.820 |
| Protocol1:WindowC | 2.687 | 0.422 | 30.239 | 6.371 | 1.826 | 3.549 |
| Protocol2:WindowC | 2.317 | 0.467 | 42.477 | 4.963 | 1.375 | 3.258 |
| Protocol3:WindowC | 2.769 | 0.451 | 38.188 | 6.144 | 1.857 | 3.682 |
| Protocol4:WindowC | 2.193 | 0.452 | 38.985 | 4.851 | 1.279 | 3.108 |
|  |  |  |  |  |  |  |
| **Global RSFC** | | | | | | |
|  | Estimate | Std. Error | DF | t value | lower | upper |
| Protocol1 | 0.578 | 0.053 | 23.821 | 10.826 | 0.468 | 0.689 |
| Protocol2 | 0.448 | 0.057 | 27.174 | 7.916 | 0.332 | 0.564 |
| Protocol3 | 0.677 | 0.056 | 27.705 | 12.008 | 0.561 | 0.793 |
| Protocol4 | 0.340 | 0.054 | 24.022 | 6.334 | 0.229 | 0.450 |
| WindowA | 0.558 | 0.032 | 46.049 | 17.585 | 0.494 | 0.622 |
| WindowB | 0.471 | 0.030 | 38.145 | 15.446 | 0.409 | 0.533 |
| WindowC | 0.503 | 0.035 | 58.937 | 14.521 | 0.434 | 0.573 |
| Protocol1:WindowA | 0.591 | 0.065 | 51.046 | 9.088 | 0.460 | 0.721 |
| Protocol2:WindowA | 0.436 | 0.063 | 42.943 | 6.965 | 0.310 | 0.562 |
| Protocol3:WindowA | 0.840 | 0.067 | 55.893 | 12.557 | 0.706 | 0.973 |
| Protocol4:WindowA | 0.365 | 0.059 | 35.260 | 6.180 | 0.245 | 0.484 |
| Protocol1:WindowB | 0.566 | 0.058 | 33.384 | 9.740 | 0.448 | 0.685 |
| Protocol2:WindowB | 0.364 | 0.065 | 43.967 | 5.635 | 0.234 | 0.494 |
| Protocol3:WindowB | 0.631 | 0.063 | 41.857 | 10.054 | 0.504 | 0.757 |
| Protocol4:WindowB | 0.323 | 0.058 | 33.352 | 5.546 | 0.204 | 0.441 |
| Protocol1:WindowC | 0.578 | 0.063 | 45.738 | 9.103 | 0.450 | 0.705 |
| Protocol2:WindowC | 0.544 | 0.073 | 66.144 | 7.420 | 0.398 | 0.690 |
| Protocol3:WindowC | 0.561 | 0.071 | 61.194 | 7.927 | 0.419 | 0.702 |
| Protocol4:WindowC | 0.331 | 0.069 | 62.488 | 4.770 | 0.192 | 0.470 |

## Supplementary Table S11. Pair-wise comparisons for Peak ΔBOLD

The tested pair-wise contrasts (output of the *difflsmeans* function from the *lmerTest* R package). Both un-adjusted and multiple-comparisons-corrected p-values (Holm method) are shown. Significant effects (corrected p<0.05) are shaded in grey. A: early (1 – 2 h); B: middle (2 – 4 h); C: late (4 – 6 h); DF: degrees of freedom with Satterthwaite's method; lower and upper: bounds of the 95% confidence interval.

| **Peak ΔBOLD** | | | | | | | | |
| --- | --- | --- | --- | --- | --- | --- | --- | --- |
| Comparison | Estimate | Std. Error | DF | t value | lower | upper | p value | p (Holm) |
| Protocol1 - Protocol2 | 0.625 | 0.566 | 24.242 | 1.104 | -0.543 | 1.793 | 0.2803 | 1.0000 |
| Protocol1 - Protocol3 | -0.348 | 0.563 | 23.933 | -0.619 | -1.510 | 0.814 | 0.5420 | 1.0000 |
| Protocol1 - Protocol4 | -0.123 | 0.559 | 23.378 | -0.219 | -1.278 | 1.033 | 0.8284 | 1.0000 |
| Protocol2 - Protocol3 | -0.973 | 0.573 | 25.374 | -1.698 | -2.153 | 0.207 | 0.1018 | 1.0000 |
| Protocol2 - Protocol4 | -0.748 | 0.570 | 24.811 | -1.313 | -1.922 | 0.426 | 0.2013 | 1.0000 |
| Protocol3 - Protocol4 | 0.226 | 0.566 | 24.502 | 0.398 | -0.942 | 1.394 | 0.6939 | 1.0000 |
| WindowA - WindowB | 1.025 | 0.133 | 262.082 | 7.712 | 0.763 | 1.286 | **<0.0001** | **<0.0001** |
| WindowA - WindowC | 1.143 | 0.153 | 266.447 | 7.462 | 0.842 | 1.445 | **<0.0001** | **<0.0001** |
| WindowB - WindowC | 0.119 | 0.140 | 262.528 | 0.849 | -0.157 | 0.394 | 0.3968 | 1.0000 |
| 1:A - 2:A | 1.218 | 0.604 | 31.600 | 2.018 | -0.012 | 2.448 | 0.0522 | 1.0000 |
| 1:A - 3:A | -0.763 | 0.615 | 34.207 | -1.240 | -2.013 | 0.487 | 0.2235 | 1.0000 |
| 1:A - 4:A | -0.416 | 0.597 | 30.450 | -0.697 | -1.635 | 0.803 | 0.4910 | 1.0000 |
| 1:A - 1:B | 1.124 | 0.248 | 259.312 | 4.535 | 0.636 | 1.612 | **<0.0001** | **0.0003** |
| 1:A - 1:C | 0.957 | 0.270 | 260.705 | 3.549 | 0.426 | 1.489 | **0.0005** | **0.0147** |
| 2:A - 3:A | -1.981 | 0.616 | 34.151 | -3.217 | -3.232 | -0.729 | 0.0028 | 0.0879 |
| 2:A - 4:A | -1.634 | 0.598 | 30.406 | -2.734 | -2.854 | -0.414 | 0.0103 | 0.2996 |
| 2:A - 2:B | 0.193 | 0.277 | 266.863 | 0.698 | -0.352 | 0.738 | 0.4860 | 1.0000 |
| 2:A - 2:C | 0.110 | 0.325 | 270.701 | 0.339 | -0.530 | 0.751 | 0.7349 | 1.0000 |
| 3:A - 4:A | 0.346 | 0.610 | 32.969 | 0.568 | -0.894 | 1.587 | 0.5737 | 1.0000 |
| 3:A - 3:B | 1.687 | 0.289 | 260.618 | 5.841 | 1.118 | 2.256 | **<0.0001** | **<0.0001** |
| 3:A - 3:C | 1.638 | 0.326 | 265.276 | 5.020 | 0.996 | 2.281 | **<0.0001** | **<0.0001** |
| 4:A - 4:B | 1.095 | 0.247 | 260.451 | 4.435 | 0.609 | 1.581 | **<0.0001** | **0.0004** |
| 4:A - 4:C | 1.868 | 0.301 | 266.864 | 6.206 | 1.275 | 2.461 | **<0.0001** | **<0.0001** |
| 1:B - 2:B | 0.287 | 0.597 | 29.835 | 0.480 | -0.933 | 1.507 | 0.6344 | 1.0000 |
| 1:B - 3:B | -0.200 | 0.587 | 28.290 | -0.340 | -1.401 | 1.002 | 0.7361 | 1.0000 |
| 1:B - 4:B | -0.445 | 0.584 | 27.834 | -0.763 | -1.642 | 0.751 | 0.4520 | 1.0000 |
| 1:B - 1:C | -0.167 | 0.240 | 260.708 | -0.695 | -0.639 | 0.305 | 0.4876 | 1.0000 |
| 2:B - 3:B | -0.487 | 0.606 | 31.463 | -0.803 | -1.722 | 0.749 | 0.4280 | 1.0000 |
| 2:B - 4:B | -0.732 | 0.603 | 30.996 | -1.214 | -1.963 | 0.498 | 0.2339 | 1.0000 |
| 2:B - 2:C | -0.083 | 0.304 | 262.342 | -0.273 | -0.681 | 0.515 | 0.7851 | 1.0000 |
| 3:B - 4:B | -0.246 | 0.593 | 29.435 | -0.414 | -1.458 | 0.966 | 0.6816 | 1.0000 |
| 3:B - 3:C | -0.049 | 0.282 | 262.238 | -0.173 | -0.604 | 0.506 | 0.8627 | 1.0000 |
| 4:B - 4:C | 0.773 | 0.289 | 264.212 | 2.675 | 0.204 | 1.342 | 0.0079 | 0.2381 |
| 1:C - 2:C | 0.371 | 0.629 | 36.237 | 0.589 | -0.905 | 1.646 | 0.5593 | 1.0000 |
| 1:C - 3:C | -0.082 | 0.617 | 34.145 | -0.133 | -1.336 | 1.172 | 0.8952 | 1.0000 |
| 1:C - 4:C | 0.494 | 0.618 | 34.519 | 0.799 | -0.762 | 1.750 | 0.4297 | 1.0000 |
| 2:C - 3:C | -0.453 | 0.649 | 40.326 | -0.698 | -1.764 | 0.858 | 0.4894 | 1.0000 |
| 2:C - 4:C | 0.123 | 0.650 | 40.733 | 0.190 | -1.189 | 1.436 | 0.8504 | 1.0000 |
| 3:C - 4:C | 0.576 | 0.638 | 38.585 | 0.902 | -0.716 | 1.868 | 0.3725 | 1.0000 |

## Supplementary Table S12. Pair-wise comparisons for Global RSFC

The tested pair-wise contrasts (output of the *difflsmeans* function from the *lmerTest* R package). Both un-adjusted and multiple-comparisons-corrected p-values (Holm method) are shown. Significant effects (corrected p<0.05) are shaded in grey. A: early (1 – 2 h); B: middle (2 – 4 h); C: late (4 – 6 h); DF: degrees of freedom with Satterthwaite's method; lower and upper: bounds of the 95% confidence interval.

| **Global RSFC** | | | | | | | | |
| --- | --- | --- | --- | --- | --- | --- | --- | --- |
| Comparison | Estimate | Std. Error | DF | t value | lower | upper | p value | p (Holm) |
| Protocol1 - Protocol2 | 0.131 | 0.078 | 25.513 | 1.677 | -0.030 | 0.291 | 0.1057 | 1.0000 |
| Protocol1 - Protocol3 | -0.099 | 0.078 | 25.761 | -1.270 | -0.258 | 0.061 | 0.2154 | 1.0000 |
| Protocol1 - Protocol4 | 0.239 | 0.076 | 23.921 | 3.155 | 0.083 | 0.395 | 0.0043 | 0.1373 |
| Protocol2 - Protocol3 | -0.229 | 0.080 | 27.437 | -2.869 | -0.393 | -0.065 | 0.0078 | 0.2192 |
| Protocol2 - Protocol4 | 0.108 | 0.078 | 25.612 | 1.389 | -0.052 | 0.269 | 0.1767 | 1.0000 |
| Protocol3 - Protocol4 | 0.337 | 0.078 | 25.861 | 4.337 | 0.177 | 0.497 | **0.0002** | **0.0070** |
| WindowA - WindowB | 0.087 | 0.027 | 277.667 | 3.207 | 0.034 | 0.140 | **0.0015** | **0.0495** |
| WindowA - WindowC | 0.054 | 0.032 | 284.369 | 1.714 | -0.008 | 0.117 | 0.0876 | 1.0000 |
| WindowB - WindowC | -0.032 | 0.029 | 278.811 | -1.106 | -0.090 | 0.025 | 0.2698 | 1.0000 |
| 1:A - 2:A | 0.155 | 0.090 | 46.903 | 1.718 | -0.027 | 0.337 | 0.0924 | 1.0000 |
| 1:A - 3:A | -0.249 | 0.093 | 53.461 | -2.666 | -0.436 | -0.062 | 0.0101 | 0.2739 |
| 1:A - 4:A | 0.226 | 0.088 | 42.890 | 2.579 | 0.049 | 0.403 | 0.0134 | 0.3360 |
| 1:A - 1:B | 0.024 | 0.054 | 273.364 | 0.454 | -0.082 | 0.131 | 0.6502 | 1.0000 |
| 1:A - 1:C | 0.013 | 0.060 | 279.485 | 0.221 | -0.105 | 0.132 | 0.8252 | 1.0000 |
| 2:A - 3:A | -0.404 | 0.092 | 49.249 | -4.407 | -0.588 | -0.220 | **0.0001** | **0.0021** |
| 2:A - 4:A | 0.071 | 0.086 | 39.060 | 0.829 | -0.103 | 0.245 | 0.4119 | 1.0000 |
| 2:A - 2:B | 0.072 | 0.056 | 283.713 | 1.288 | -0.038 | 0.182 | 0.1989 | 1.0000 |
| 2:A - 2:C | -0.108 | 0.066 | 290.319 | -1.630 | -0.238 | 0.022 | 0.1042 | 1.0000 |
| 3:A - 4:A | 0.475 | 0.089 | 45.196 | 5.327 | 0.295 | 0.654 | **<0.0001** | **0.0001** |
| 3:A - 3:B | 0.209 | 0.059 | 276.898 | 3.530 | 0.092 | 0.325 | **0.0005** | **0.0170** |
| 3:A - 3:C | 0.279 | 0.067 | 283.239 | 4.161 | 0.147 | 0.410 | **<0.0001** | **0.0016** |
| 4:A - 4:B | 0.042 | 0.047 | 274.800 | 0.895 | -0.050 | 0.134 | 0.3717 | 1.0000 |
| 4:A - 4:C | 0.033 | 0.060 | 281.500 | 0.556 | -0.085 | 0.151 | 0.5785 | 1.0000 |
| 1:B - 2:B | 0.203 | 0.087 | 38.709 | 2.333 | 0.027 | 0.378 | 0.0249 | 0.5733 |
| 1:B - 3:B | -0.064 | 0.086 | 37.599 | -0.750 | -0.237 | 0.109 | 0.4577 | 1.0000 |
| 1:B - 4:B | 0.244 | 0.082 | 33.368 | 2.960 | 0.076 | 0.411 | 0.0056 | 0.1630 |
| 1:B - 1:C | -0.011 | 0.052 | 278.462 | -0.215 | -0.114 | 0.091 | 0.8298 | 1.0000 |
| 2:B - 3:B | -0.267 | 0.090 | 42.924 | -2.966 | -0.448 | -0.085 | 0.0049 | 0.1511 |
| 2:B - 4:B | 0.041 | 0.087 | 38.685 | 0.470 | -0.135 | 0.217 | 0.6407 | 1.0000 |
| 2:B - 2:C | -0.180 | 0.063 | 279.746 | -2.838 | -0.305 | -0.055 | 0.0049 | 0.1511 |
| 3:B - 4:B | 0.308 | 0.086 | 37.577 | 3.597 | 0.134 | 0.481 | **0.0009** | **0.0314** |
| 3:B - 3:C | 0.070 | 0.060 | 276.482 | 1.158 | -0.049 | 0.188 | 0.2479 | 1.0000 |
| 4:B - 4:C | -0.008 | 0.059 | 280.335 | -0.143 | -0.124 | 0.107 | 0.8863 | 1.0000 |
| 1:C - 2:C | 0.034 | 0.097 | 56.112 | 0.348 | -0.160 | 0.228 | 0.7290 | 1.0000 |
| 1:C - 3:C | 0.017 | 0.095 | 53.526 | 0.177 | -0.174 | 0.207 | 0.8604 | 1.0000 |
| 1:C - 4:C | 0.246 | 0.094 | 53.962 | 2.619 | 0.058 | 0.435 | 0.0114 | 0.2968 |
| 2:C - 3:C | -0.017 | 0.102 | 63.691 | -0.166 | -0.220 | 0.187 | 0.8683 | 1.0000 |
| 2:C - 4:C | 0.213 | 0.101 | 64.379 | 2.106 | 0.011 | 0.414 | 0.0391 | 0.8604 |
| 3:C - 4:C | 0.230 | 0.099 | 61.824 | 2.316 | 0.031 | 0.428 | 0.0239 | 0.5733 |

# References

1. Weber, R., Ramos-Cabrer, P., Wiedermann, D., Van Camp, N. & Hoehn, M. A fully noninvasive and robust experimental protocol for longitudinal fMRI studies in the rat. *Neuroimage* **29**, 1303–1310 (2006).

2. Weber, R. *et al.* Early Prediction of Functional Recovery after Experimental Stroke: Functional Magnetic Resonance Imaging, Electrophysiology, and Behavioral Testing in Rats. *J. Neurosci.* **28**, 1022–1029 (2008).

3. Zhao, F., Zhao, T., Zhou, L., Wu, Q. & Hu, X. BOLD study of stimulation-induced neural activity and resting-state connectivity in medetomidine-sedated rat. *Neuroimage* **39**, 248–260 (2008).

4. Pawela, C. P. *et al.* Resting-state functional connectivity of the rat brain. *Magn. Reson. Med.* **59**, 1021–1029 (2008).

5. Pawela, C. P. *et al.* A protocol for use of medetomidine anesthesia in rats for extended studies using task-induced BOLD contrast and resting-state functional connectivity. *Neuroimage* **46**, 1137–1147 (2009).

6. Seehafer, J. U., Kalthoff, D., Farr, T. D., Wiedermann, D. & Hoehn, M. No Increase of the Blood Oxygenation Level-Dependent Functional Magnetic Resonance Imaging Signal with Higher Field Strength: Implications for Brain Activation Studies. *J. Neurosci.* **30**, 5234–5241 (2010).

7. Williams, K. A. *et al.* Comparison of alpha-chloralose, medetomidine and isoflurane anesthesia for functional connectivity mapping in the rat. *Magn. Reson. Imaging* **28**, 995–1003 (2010).

8. Angenstein, F., Krautwald, K. & Scheich, H. The current functional state of local neuronal circuits controls the magnitude of a BOLD response to incoming stimuli. *Neuroimage* **50**, 1364–1375 (2010).

9. Airaksinen, A. M. *et al.* Simultaneous fMRI and local field potential measurements during epileptic seizures in medetomidine-sedated rats using raser pulse sequence. *Magn. Reson. Med.* **64**, 1191–9 (2010).

10. Jonckers, E., van Audekerke, J., de Visscher, G., van der Linden, A. & Verhoye, M. Functional connectivity fMRI of the rodent brain: Comparison of functional connectivity networks in rat and mouse. *PLoS One* **6**, (2011).

11. Majeed, W. *et al.* Spatiotemporal dynamics of low frequency BOLD fluctuations in rats and humans. *Neuroimage* **54**, 1140–1150 (2011).

12. Airaksinen, A. M. *et al.* Simultaneous BOLD fMRI and local field potential measurements during kainic acid-induced seizures. *Epilepsia* **53**, 1245–1253 (2012).

13. Krautwald, K. & Angenstein, F. Low Frequency Stimulation of the Perforant Pathway Generates Anesthesia-Specific Variations in Neural Activity and BOLD Responses in the Rat Dentate Gyrus. *J. Cereb. Blood Flow Metab.* **32**, 291–305 (2012).

14. Ciobanu, L., Reynaud, O., Uhrig, L., Jarraya, B. & Le Bihan, D. Effects of anesthetic agents on brain blood oxygenation level revealed with ultra-high field MRI. *PLoS One* **7**, e32645 (2012).

15. Nasrallah, F. A., Tan, J. & Chuang, K.-H. H. Pharmacological modulation of functional connectivity: α2-adrenergic receptor agonist alters synchrony but not neural activation. *Neuroimage* **60**, 436–446 (2012).

16. Kalthoff, D., Po, C., Wiedermann, D. & Hoehn, M. Reliability and spatial specificity of rat brain sensorimotor functional connectivity networks are superior under sedation compared with general anesthesia. *NMR Biomed.* **26**, 638–650 (2013).

17. Schwarz, A. J. *et al.* Anti-Correlated Cortical Networks of Intrinsic Connectivity in the Rat Brain. *Brain Connect.* **3**, 503–511 (2013).

18. Angenstein, F., Krautwald, K., Wetzel, W. & Scheich, H. Perforant pathway stimulation as a conditioned stimulus for active avoidance learning triggers BOLD responses in various target regions of the hippocampus: A combined fMRI and electrophysiological study. *Neuroimage* **75**, 213–227 (2013).

19. Sekar, S. *et al.* Subchronic memantine induced concurrent functional disconnectivity and altered ultra-structural tissue integrity in the rodent brain: Revealed by multimodal MRI. *Psychopharmacology (Berl).* **227**, 479–491 (2013).

20. Uhrig, L., Ciobanu, L., Djemai, B., Le Bihan, D. & Jarraya, B. Sedation agents differentially modulate cortical and subcortical blood oxygenation: evidence from ultra-high field MRI at 17.2 T. *PLoS One* **9**, e100323 (2014).

21. Nasrallah, F. A., Lew, S. K., Low, A. S.-M. & Chuang, K.-H. Neural correlate of resting-state functional connectivity under α2 adrenergic receptor agonist, medetomidine. *Neuroimage* **84**, 27–34 (2014).

22. D’Souza, D. V. *et al.* Preserved Modular Network Organization in the Sedated Rat Brain. *PLoS One* **9**, e106156 (2014).

23. Duffy, B. A., Choy, M., Chuapoco, M. R., Madsen, M. & Lee, J. H. MRI compatible optrodes for simultaneous LFP and optogenetic fMRI investigation of seizure-like afterdischarges. *Neuroimage* **123**, 173–184 (2015).

24. Sierakowiak, A. *et al.* Default mode network, motor network, dorsal and ventral basal ganglia networks in the rat brain: comparison to human networks using resting state-fMRI. *PLoS One* **10**, e0120345 (2015).

25. Nasrallah, F. A., To, X. V., Chen, D. Y., Routtenberg, A. & Chuang, K. H. Functional connectivity MRI tracks memory networks after maze learning in rodents. *Neuroimage* **127**, 196–202 (2016).

26. Paasonen, J., Salo, R. A., Huttunen, J. K. & Grohn, O. Resting-state functional MRI as a tool for evaluating brain hemodynamic responsiveness to external stimuli in rats. *Magn. Reson. Med* **78**, 1136–1146 (2016).

27. Scherf, T. & Angenstein, F. Hippocampal CA3 activation alleviates fMRI-BOLD responses in the rat prefrontal cortex induced by electrical VTA stimulation. *PLoS One* **12**, e0172926 (2017).

28. Albers, F., Schmid, F., Wachsmuth, L. & Faber, C. Line scanning fMRI reveals earlier onset of optogenetically evoked BOLD response in rat somatosensory cortex as compared to sensory stimulation. *Neuroimage* **164**, 144–154 (2018).

29. Wang, M., He, Y., Sejnowski, T. J. & Yu, X. Brain-state dependent astrocytic Ca2+ signals are coupled to both positive and negative BOLD-fMRI signals. *Proc. Natl. Acad. Sci. U. S. A.* **115**, E1647–E1656 (2018).

30. Shatillo, A. *et al.* Spontaneous BOLD waves – A novel hemodynamic activity in Sprague-Dawley rat brain detected by functional magnetic resonance imaging. *J. Cereb. Blood Flow Metab* (2018). doi: 10.1177/0271678X18772994

31. Paasonen, J., Stenroos, P., Salo, R. A., Kiviniemi, V. & Gröhn, O. Functional connectivity under six anesthesia protocols and the awake condition in rat brain. *Neuroimage* **172**, 9–20 (2018).

32. Fukuda, M., Vazquez, A. L., Zong, X. & Kim, S.-G. G. Effects of the alpha(2)-adrenergic receptor agonist dexmedetomidine on neural, vascular and BOLD fMRI responses in the somatosensory cortex. *Eur. J. Neurosci.* **37**, 80–95 (2013).

33. Pan, W.-J., Thompson, G. J., Magnuson, M. E., Jaeger, D. & Keilholz, S. Infraslow LFP correlates to resting-state fMRI BOLD signals. *Neuroimage* **74**, 288–297 (2013).

34. Chao, T. H. H., Chen, J. H. & Yen, C. T. Repeated BOLD-fMRI imaging of deep brain stimulation responses in rats. *PLoS One* **9**, (2014).

35. Magnuson, M. E., Thompson, G. J., Pan, W. J. & Keilholz, S. D. Time-dependent effects of isoflurane and dexmedetomidine on functional connectivity, spectral characteristics, and spatial distribution of spontaneous BOLD fluctuations. *NMR Biomed.* **27**, 291–303 (2014).

36. Magnuson, M. E., Thompson, G. J., Pan, W.-J. & Keilholz, S. D. Effects of severing the corpus callosum on electrical and BOLD functional connectivity and spontaneous dynamic activity in the rat brain. *Brain Connect.* **4**, 15–29 (2014).

37. Li, N., Van Zijl, P., Thakor, N. & Pelled, G. Study of the spatial correlation between neuronal activity and BOLD fMRI responses evoked by sensory and channelrhodopsin-2 stimulation in the rat somatosensory cortex. *J. Mol. Neurosci.* **53**, 553–561 (2014).

38. Thompson, G. J. *et al.* Phase-amplitude coupling and infraslow (<1 Hz) frequencies in the rat brain: relationship to resting state fMRI. *Front. Integr. Neurosci.* **8**, 41 (2014).

39. Thompson, G. J., Pan, W.-J., Magnuson, M. E., Jaeger, D. & Keilholz, S. D. Quasi-periodic patterns (QPP): large-scale dynamics in resting state fMRI that correlate with local infraslow electrical activity. *Neuroimage* **84**, 1018–1031 (2014).

40. Medda, A. *et al.* Wavelet-based clustering of resting state MRI data in the rat. *Magn. Reson. Imaging* **34**, 35–43 (2016).

41. Power, J. D., Barnes, K. A., Snyder, A. Z., Schlaggar, B. L. & Petersen, S. E. Spurious but systematic correlations in functional connectivity MRI networks arise from subject motion. *Neuroimage* **59**, 2142–2154 (2012).
